# Optimization of nitric oxide donors for investigating biofilm dispersal response in *Pseudomonas aeruginosa* clinical isolates

**DOI:** 10.1007/s00253-020-10859-7

**Published:** 2020-08-31

**Authors:** Yu-ming Cai, Jeremy S. Webb

**Affiliations:** Institute for Life Sciences, University of Southampton, Southampton, SO17 1BJ UK

**Keywords:** Nitric oxide, *Pseudomonas aeruginosa*, Biofilm, Cystic fibrosis, Chemiluminescence

## Abstract

**Abstract:**

*Pseudomonas aeruginosa* biofilms contribute heavily to chronic lung infection in cystic fibrosis patients, leading to morbidity and mortality. Nitric oxide (NO) has been shown to disperse *P. aeruginosa* biofilms in vitro, ex vivo and in clinical trials as a promising anti-biofilm agent. Traditional NO donors such as sodium nitroprusside (SNP) have been extensively employed in different studies. However, the dosage of SNP in different studies was not consistent, ranging from 500 nM to 500 μM. SNP is light sensitive and produces cyanide, which may lead to data misinterpretation and inaccurate predictions of dispersal responses in clinical settings. New NO donors and NO delivery methods have therefore been explored. Here we assessed 7 NO donors using *P. aeruginosa* PAO1 and determined that SNP and Spermine NONOate (S150) successfully reduced > 60% biomass within 24 and 2 h, respectively. While neither dosage posed toxicity towards bacterial cells, chemiluminescence assays showed that SNP only released NO upon light exposure in M9 media and S150 delivered much higher performance spontaneously. S150 was then tested on 13 different cystic fibrosis *P. aeruginosa* (CF-PA) isolates; most CF-PA biofilms were significantly dispersed by 250 μM S150. Our work therefore discovered a commercially available NO donor S150, which disperses CF-PA biofilms efficiently within a short period of time and without releasing cyanide, as an alternative of SNP in clinical trials in the future.

**Key points:**

• *S150 performs the best in dispersing P. aeruginosa biofilms among 7 NO donors.*

• *SNP only releases NO in the presence of light, while S150 releases NO spontaneously.*

• *S150 successfully disperses biofilms formed by P. aeruginosa cystic fibrosis clinical isolates.*

**Electronic supplementary material:**

The online version of this article (10.1007/s00253-020-10859-7) contains supplementary material, which is available to authorized users.

## Introduction

The major cause of morbidity and mortality in cystic fibrosis (CF) patients is the chronic bacterial colonization of patients’ lungs and airways leading to pulmonary dysfunction and infection (Gilligan 1991; Govan and Deretic 1996). When bacteria invade healthy individuals, opportunistic pathogens that overcome mucociliary clearance can be targeted by phagocytic cells and specific opsonizing antibodies (Govan and Deretic 1996). However, in CF patients, the dehydrated surface liquid on respiratory epithelium results in defective mucociliary clearance and frustrated phagocytosis due to the impaired opsonisation process, hence contributing to the chronic colonization (Govan and Deretic 1996). *Pseudomonas aeruginosa* has been well recognized as the most commonly found and important pathogen in progressive and severe CF lung disease. CF *P. aeruginosa* clinical isolates frequently show higher levels of persister cells and antibiotic resistance (Saiman et al. 1996; Mulcahy et al. 2010). Furthermore, *P. aeruginosa* tend to form aggregates/biofilms in vivo, leading to a much higher tolerance to treatments, driven by both the protective extracellular polymeric substances (EPS) produced by bacteria and local host environment (Ciofu and Tolker-Nielsen 2019). Once biofilms are established, they are almost impossible to eradicate (Høiby et al. 2005).

One promising strategy is to combine biofilm dispersal agents and conventional antibiotics, where bacterial cells become more susceptible once reversed back to planktonic form. Nitric oxide (NO) was discovered to be an anti-biofilm signalling molecule and the mechanisms are still under investigation (Barraud et al. 2006). In *P. aeruginosa*, biofilm phenotypes have been associated with an important secondary messenger, cyclic-di-GMP (c-di-GMP), which contributes to a myriad of physiological changes and the establishment of biofilms. A decrease in intracellular c-di-GMP level promotes motile mode of growth of bacteria and triggers biofilm dispersal (Ute Römling et al. 2013). NO was demonstrated to reduce c-di-GMP level by stimulating the activities of phosphodiesterases responsible for the hydrolysis of c-di-GMP, upregulate genes involved in motility and downregulate those related to the expression of adhesins and virulence factors (Rinaldo et al. 2018; Barraud et al. 2009b). Although NO can be endogenously generated by *P. aeruginosa*, which facilitates biofilm dispersal at the late stage of biofilm life cycle, exogenously added NO significantly accelerates the procedure and can also prevent the early attachment. Previous studies reported that the efficacies of conventional antibiotics towards established biofilms were significantly enhanced when NO was applied as a dispersal agent (Barraud et al. 2006; Howlin et al. 2011; Soren et al. 2019). NO gas has also been applied in clinical trials, where it increased the efficacy of conventional antibiotics in CF lung infection treatment (Howlin et al. 2017; Cathie et al. 2014). Therefore, NO has been regarded as a putative anti-biofilm adjunctive therapy. Conventional NO donors such as sodium nitroprusside (SNP), S-Nitrosothiols (RSNOs) and Diazeniumdiolates (NONOates) have been applied as dispersal agents against biofilms formed by different clinically relevant bacterial species such as *P. aeruginosa, Escherichia coli, Neisseria gonorrhoeae, Staphylococcus aureus* and *Staphylococcus epidermidis* (Barraud et al. 2006; Barraud et al. 2009a; De La Fuente-Núñez et al. 2013; Barnes et al. 2013; Sulemankhil et al. 2012; Falsetta et al. 2009; Jardeleza et al. 2011). Along with these, many studies were also carried out to explore novel methods for NO delivery, such as incorporating NO donors into nanoparticles and polymer coating (Sadrearhami et al. 2017; Duong et al. 2014a; Nablo and Schoenfisch 2003; Nablo et al. 2005). However, more often than not these studies tested different concentrations of NO donors towards early stage biofilms formed by type strains, or did not specify optimal treatment time which is crucial for the interpretation of data from young biofilms to distinguish between prevention and dispersal (Barraud et al. 2009a; Barnes et al. 2013; Sadrearhami et al. 2017; Duong et al. 2014a; Shen et al. 2019; Duong et al. 2014b; Zhu et al. 2018; Marvasi et al. 2014). To this end, we systematically compared the optimal concentrations and treatment time of 7 NO donors for triggering biofilm dispersal using *P. aeruginosa* PAO1, including SNP, S-nitroso-glutathione (GSNO), S-nitroso-N-acetyl-DL-penicillamine (SNAP), 1-(hydroxy-NNO-azoxy)-L-proline (PROLI NONOate), 6-(2-hydroxy-1-methyl-2-nitrosohydrazino)-N-methyl-1-hexanamine (MAHMA NONOate), (Z)-1-[N-[3-aminopropyl]-N-[4-(3-aminopropylammonio)butyl]-amino]diazen-1-ium-1,2-diolate (Spermine NONOate) and diethylammonium (Z)-1-(N,N-diethylamino)diazen-1-ium-1,2-diolate (DEA-NONOate). The selection was based on different categories of NO donors previously reported to be used in laboratories, animal experiments or clinical settings with different NO release mechanisms. While SNP has been traditionally and widely applied, the mechanism for NO release is very complicated depending on conditions and involves three stages (Smith and Dasgupta 2002). GSNO and SNAP belong to S-nitrosothiols, a class of NO donor releasing NO and nitrosonium (NO^+^) spontaneously from the moiety (-SNO) (Napoli and Ignarro 2003). Both GSNO and SNAP were initially investigated for their role as antiplatelet agents in cardiovascular system, as S-nitrosothiols do not appear to engender vascular tolerance (Belcastro et al. 2017; Brisbois et al. 2013). In contrast, NONOates, i.e. diazeniumdiolates, consist of a diolate group [N(O-)N=O] bound to a nucleophile adduct via a nitrogen atom (Maragos et al. 1991). Although not yet approved for clinical use so far, NONOate is one of the most investigated NO donors due to its capability to release two moles of NO per mole of donor at physiological conditions, showing outstanding clinical application prospects (Yang et al. 2018; Li et al. 2020). As such, four different NONOates previously investigated in other biofilm studies (Barnes et al. 2013; Barnes et al. 2015; Zhu et al. 2018; Marvasi et al. 2014) were chosen in this study. After a high-throughput screening, both 250 μM SNP and Spermine NONOate (S150) showed great dispersal efficacies after 24 h and 2 h, respectively. However, by employing a highly sensitive chemiluminescence detection method, we confirmed that S150 exhibited better performance in releasing NO and is more suitable for testing biofilm dispersal response to NO.

As CF-PAs can undergo genetic adaption catalysed by hypermutation in chronic infection lungs (Mena et al. 2008; Bianconi et al. 2019; Caçador et al. 2018; Winstanley et al. 2016), it is suspected that some CF-PA isolates may contain mutations that lead to higher tolerance to NO. Using 250 μM S150, we tested the NO response of 72-h biofilms in microtiter plates formed by 13 different CF-PA isolates and most strains were successfully dispersed. In summary, our study showed that (1) S150 is superior to SNP, which can be applied in wider settings as it does not require light to release NO; (2) S150 can disperse biofilms formed by genetically different CF-PA strains, indicating its potential clinical applications.

## Materials and methods

### Ethics for cystic fibrosis patient sputum collection

Sputum samples from 72 patients with CF were obtained by CF physiotherapist-assisted sample expectoration following Good Clinical Practice guidelines (ICH) (Blau et al. 2014; Aaron et al. 2004). All sampling protocols and procedures were approved by UK NHS Research Ethics Committee (South Central – Hampshire A Research Ethics Committee, Reference 08/H0502/126, Mechanisms of lung infection and inflammation in respiratory disease). Informed consent was obtained from all subjects or, if subjects were under 18, from a parent and/or legal guardian.

### Bacterial strains and culture conditions

Bacterial strains used in this study are listed in Supplementary Table S1. All bacterial overnight cultures were grown in lysogeny broth (LB) medium for 15 h at 37 °C. For CF-PA isolation, sputa samples were digested using Mucolyse™ Sputum Digestant (Pro-Lab Diagnostics, UK) for 15 mins at 37 °C, followed by culture on *P. aeruginosa*-specific cetrimide agar (Sigma-Aldrich, UK). Multiplex PCR was used to confirm *P. aeruginosa* as previously described by De Vos et al. (1997).

### Preparation of NO donor/NO scavenger solution

NO donors with different half-lives in pH 7.4 buffer tested in this study are listed in Table 1**.** Sodium nitroprusside (SNP), S-nitroso-N-acetyl-DL-penicillamine (SNAP), S-nitrosoglutathione (GSNO), MAHMA NONOate (NOC-9), PROLI NONOate and Spermine NONOate (S150), diethylamine NONOate sodium salt hydrate (DEA NONOate) and carboxy-PTIO potassium salt (PTIO) were purchased from Sigma Aldrich, UK. SNP and RSNOs stock solutions were prepared in phosphate saline buffer (pH 7.4), with SNP prepared and kept in dark. NONOates stock solutions were prepared in 0.01 M NaOH. All stock solutions were filter sterilized and diluted into fresh M9 media on ice. During preparation procedures, all solutions were kept on ice before use and SNP was kept in the dark. All treatments were conducted at 37°C, with SNP exposed to light.

**Table 1:** NO donors tested in this study and their half-lives at 37°C, pH ~ 7.4

SNP	< 2 mins
SNAP	6 h
GSNO	1–3 h
PROLI NONOate	1.8 s
MAHMA NONOate	1 min
Spermine NONOate (S150)	39 min
DEA-NONOate	2 min

### Batch cultured biofilms

For microtiter plate–based biofilm assays, 100 μl of each culture in M9 medium (OD_600nm_ ~ 0.01) was inoculated into each well. Microtiter plates were incubated statically for 24 h, and biofilms stained with 0.1% (*w*/*v*) crystal violet after two washes, dissolved in 30% (*v*/v) acetic acid. Crystal violet staining was quantified at a wavelength of 584 nm.

For microscopic biofim examination, 3 ml of culture in M9 medium (OD_600nm_ ~ 0.01) was inoculated into a MatTek plates (P35G-1.5-14-C). Plates were shaken at 50 rpm and biofilms stained with LIVE/DEAD® BacLight (Invitrogen, UK) and examined by confocal laser scanning microscopy (CLSM). A wavelength of 488 nm was used for SYTO-9 and 561 nm was used for propidium iodide excitation. At least 3 image stacks were taken from random locations in each MatTek plate. Total biomass for biofilms in each micrograph was calculated by software COMSTAT (Heydorn et al. 2000).

For NO donor screening assay using PAO1 biofilms, NO donors were added to the 24-h pre-established biofilms in microtiter plates and then incubated for a further 1, 2, 4, 6, 8, 12 or 24 h as treatment. For NO-induced dispersal in PAO1 and CF-PA biofilms, S150 was added to 72-h pre-established biofilms in both microtiter plates and MatTek plates and then incubated at 37 °C for 2 h to trigger dispersal.

### Bactericidal test

Bactericidal test method was modified from Barnes et al. (2013). Briefly, 1 ml overnight cultures were centrifuged at 4000 × *g* for 10 mins to harvest the cells in Eppendorf tubes, washed twice in sterile PBS and re-suspended in 1 ml M9. Serial dilutions of the cells were made to approximately 10^4^ CFU/ml in M9. SNP and S150 donor stock solutions were added into the culture making a desired final concentration. Cells were then incubated at 37 °C for 2 h or 24 h in M9 before determining the final CFU following a modified Miles et al. (1938).

### NO release quantification using chemiluminescence

NO gas released by SNP and S150 in M9 medium was detected in a chemiluminescence CLD 88Y NO analyser (EcoPhysics, Durnten, Switzerland) with synthetic air (99.99999% BOC) as an inert carrier. The photomultiplier detects emissions above 600 nm, and the NO concentration was calculated from the emitted intensity against a calibration standard using NaNO_2_ as previously reported (standard curve at 250, 375, 500, 750 and 1000 pmol NO, Supplementary Fig. S1) (Piknova and Schechter 2011). Tracings were recorded at 4-Hz frequency using PowerChrom® (eDAQ Pty LtD, Australia). Data were plotted using Origin 9, calculating areas to quantify total NO release within 2 h, comparing NO donor samples against standards.

### Statistical analyses

All assays were assessed using the two-tailed Student’s *T* test. Statistical significances were *P* < 0.05 for all measurements reported unless otherwise stated. Statistics and graphs were produced using GraphPad Prism.

## Results

### SNP and S150 successfully remove PAO1 biofilms

**Fig. 1:**
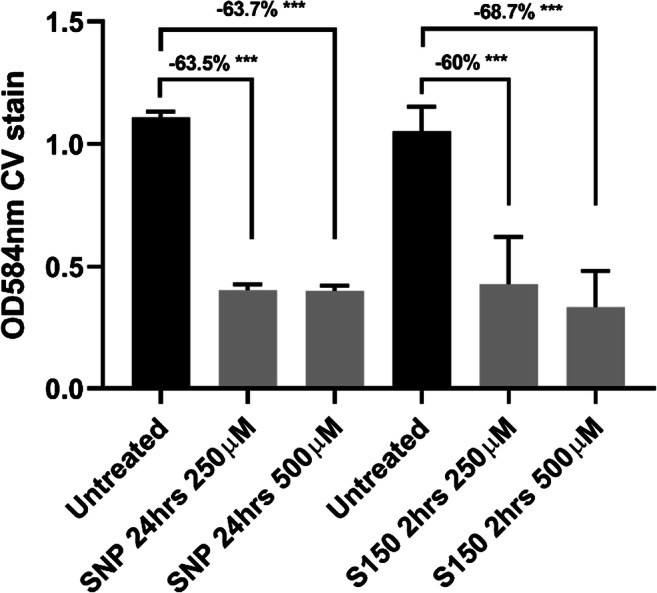
Biomass reduction of pre-established 24-h biofilms after SNP or S150 treatment (SNP treatment for 24 h, S150 for 2 h), shown by CV staining in microtiter plates. Reduction percentages were labelled above each treatment group. ** denotes 0.01 < *P* < 0.05, *** denotes *P* < 0.01. *n* = 3 independent experiments × 6 technical replicates

The efficacies of 7 donors in M9 medium at 9 concentrations (1 μM, 2.5 μM, 5 μM, 10 μM, 25 μM, 50 μM, 100 μM, 250 μM and 500 μM) and 7 treatment time periods (1 h, 2 h, 4 h, 6 h, 8 h, 12 h, 24 h) were systematically tested at 37°C in microtiter plates. All data were shown in Supplementary Fig. S2–8. Among all conditions, 24-h 250 μM SNP treatment and 2-h 250 μM S150 treatment were determined as optimal (> 60%) for triggering *P. aeruginosa* dispersal **(**Fig. 1**)**. 500μM dosage for both compounds performed comparably with 250 μM. To avoid overexposure of NO, 250 μM was selected for further tests.

### S150 exhibits higher NO release efficiency than SNP

**Fig. 2:**
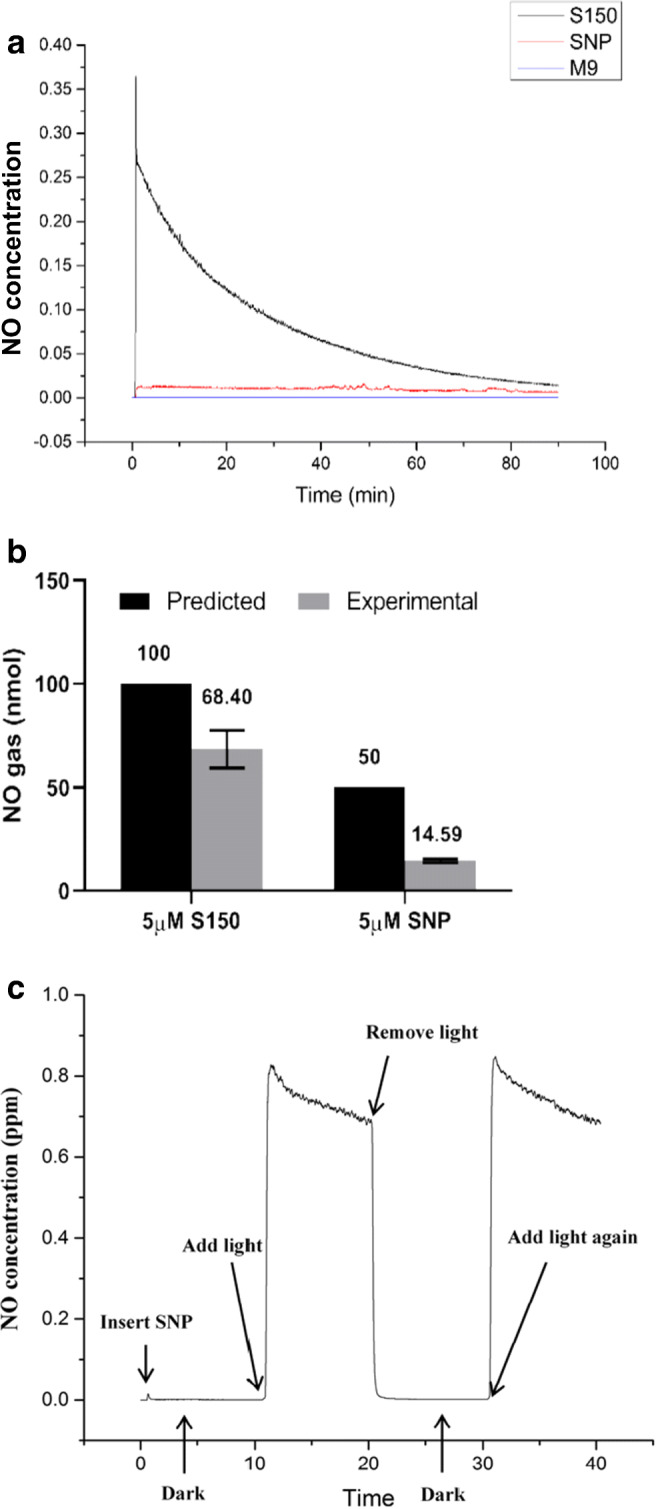
**a** NO release curves from CLD tests of 5 μM S150, 5 μM SNP and M9 media. **b** Total NO amount (nmol) released from 10 ml 5 μM S150/SNP within 1.5 h. ** denotes 0.01 < *P* < 0.05, *** denotes *P* < 0.01. **c** NO release curve from 500 μM SNP with/without cold light source at 37 °C in M9 media. *n* = 3 independent experiments

Gas phase chemiluminescence is a sensitive method to record and quantify the precise NO release, which was therefore employed in this study to compare the difference between SNP and S150. Due to the 1 ppm maximum detection limit of CLD 88Y, NO released from 5 μM S150 and SNP was quantified in M9 media at 37°C across ~ 1.5 h timeframe as shown in Fig. 2a and b**.** Results showed that at 37°C, S150 spontaneously released NO upon contact with the medium, while SNP steadily released low amount of NO. As 1 molecule of S150 releases 2 molecules of NO and SNP only releases 1, the predicted NO release from S150 is twice more than the same amount of SNP. However, the efficiency of S150 within 1.5 h was 68.4 ± 8.1%, while SNP was only 29.2 ± 2.2% **(**Fig. 2b**)**. Therefore, it can be concluded that at the same concentration, S150 is more effective in releasing NO within a relatively short time frame. As SNP degradation is associated with light exposure, a PHOTONIC PL3000 device (maximum light intensity 26 Mlx, colour temperature 3250 K) was applied for a constant cold light source without disturbing the incubation temperature. 500μM SNP was chosen for more obvious releasing curves, and foil paper was used to wrap the whole system when light was forbidden in the tests. From Fig. 2c**,** it can be concluded that SNP effectively released NO when light was present. However, SNP stopped releasing NO immediately after the light exposure was withdrawn, confirming the necessity of light for SNP as a NO donor. Even at a concentration of 500 μM, the peak NO concentration released from NO only reached 0.8 ppm, while S150 immediately released 0.35 ppm at 5 μM. Therefore, S150 was chosen for its spontaneous, more consistent and efficient performance without the production of cyanide in further assays.

### S150 triggers biofilm dispersal through NO, not cytotoxicity

**Fig. 3:**
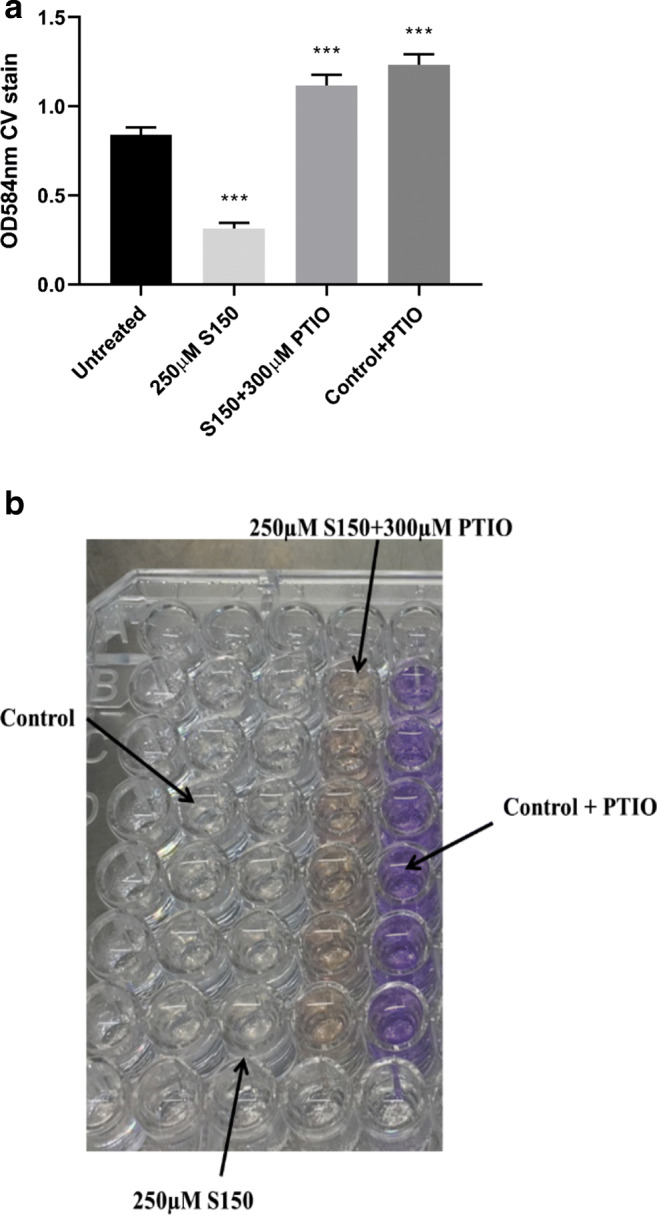
**a** Biofilm removal test of S150 with NO scavenger PTIO. **b** PAO1 biofilms in microtiter plates treated by S150 and PTIO. ** denotes 0.01 < *P* < 0.05, *** denotes *P* < 0.01. *n* = 3 independent experiments × 6 technical replicates

Previous studies have shown that NO scavenger (PTIO) can abolish the dispersal effect from SNP (Barraud et al. 2006; Howlin et al. 2017). Here, we also tested whether biofilm dispersal triggered by S150 was due to side effects or NO released from the donor using NO scavenger PTIO. Usually a higher concentration of PTIO than that of NO donor was added to ensure all NO could be scavenged (Barraud et al. 2006). As shown in Fig. 3a, 250 μM S150 triggered dispersal in 2 h, while PTIO abolished biofilm dispersal totally (biomass increased by 33.4 ± 9.7%, *P* < 0.001). For control + PTIO, a higher increase occurred (47.2 ± 11.3%). The colour of combination of S150 and PTIO turned yellow while control added in PTIO remained purple in Fig. 3b, indicating S150 was reacting with PTIO. Thus, PTIO prevented NO release from S150, and the increase of biofilm might be due to excessive PTIO inhibited NO release from nitrite reductase (Barraud et al. 2006).

**Fig. 4:**
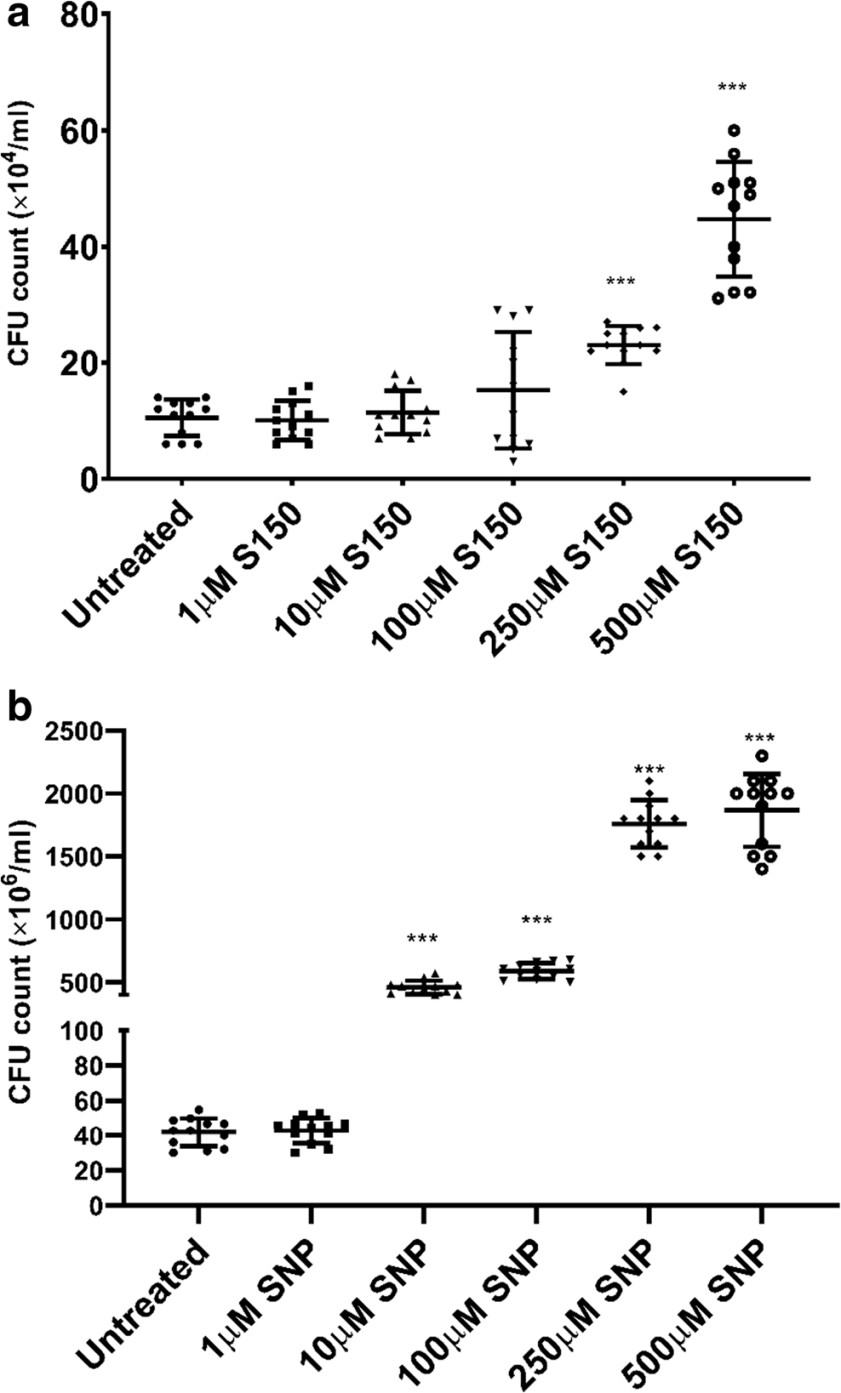
Bactericidal test for **a** different concentrations of S150 (2 h) and **b** different concentrations of SNP (24 h) on PAO1 WT planktonic cells grown in M9. ** denotes 0.01 < *P* < 0.05, *** denotes *P* < 0.01. *n* = 3 independent experiments × 4 technical replicates

After confirming it was NO triggering biofilm dispersal, we next determined if the removal of biofilms was due to the bactericidal effect. The toxicity of NO was tested for planktonic PAO1 cells in M9 media at 37°C using an initial inoculum of CFU ~ 1 × 10^4^/ml. Planktonic cultures were treated for 2 h using S150, and 24 h using SNP according to their optimal dispersal time. Figure 4 showed that both SNP and S150 surprisingly enhanced planktonic cells’ number at higher concentrations. Theoretically 250 μM S150 and 500 μM SNP should release the same amount of NO, and they increased CFU by 2.4 ± 0.6-fold (*P* < 0.001) and 46 ± 8-fold (*P* < 0.001), respectively. Whether it was NO per se or the breakdown products from donors that enhanced the growth remains unknown. Nevertheless, the long treatment time (24 h) needed for SNP contributed to much larger errors in data interpretation due to much higher increase in CFU, and S150 was therefore again proven to be superior.

### S150 successfully disperses biofilms formed by CF-PAs

**Fig. 5:**
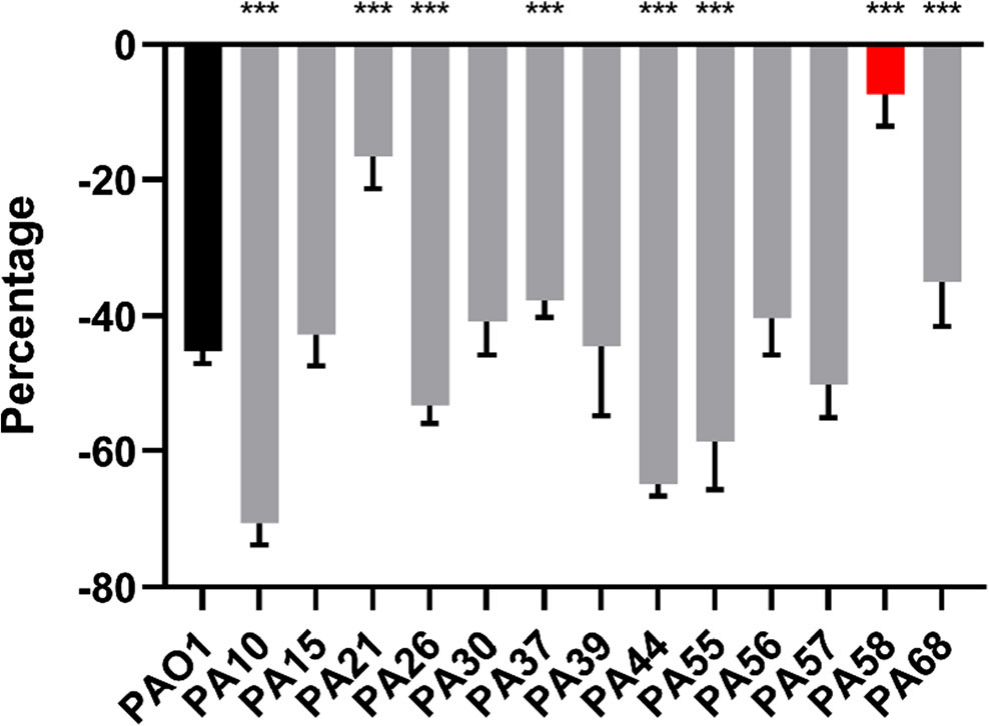
Pre-established, 72-h CF-PA biofilms treated with 250 μM S150. Biomass reduction percentage of each CF-PA was compared with PAO1. PA58 was shown as red due to the fact that the biomass difference between control and treated groups were not significant. *** denotes *P* < 0.01, ** denotes 0.01 < *P* < 0.05. *n* = 3 independent experiments × 6 technical replicates

**Fig. 6:**
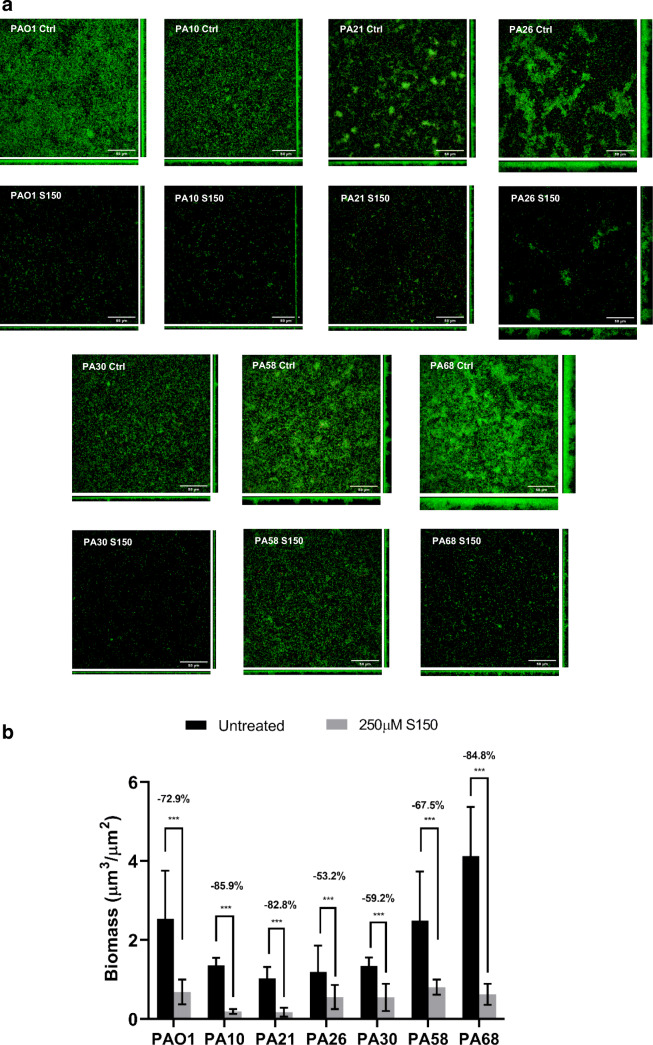
**a** Selective confocal laser scanning microscopic micrographs of 72-h PAO1, PA10, PA21, PA26, PA30, PA58 and PA68 biofilms grown at 37 °C with/without S150 treatment. Scale bar = 50 μm. **b** 72-h CF-PA biofilms with 250 μM S150 treatment. Biomass reduction was analysed by COMSTAT

As CF-PA strains often go through genetic adaptation in chronically infected lungs, we tested whether the biofilms formed by different clinical isolates can also be dispersed by optimized NO donor. However, most clinical isolates exhibit much slower growth rates and biofilm establishment compared with type strain PAO1. Previous microtiter plate screening in the lab showed that most CF-PA isolates formed the thickest biofilms at 72 h, after which the automatic dispersal stage began (data not shown). S150 treatment was also carried out on 24-h, 48-h, 72-h, 96-h, and 120-h PAO1 biofilms, and the results showed that the biomass reduction of 72-h PAO1 biofilms after S150 treatment was around 40–45%. As such, to test S150 on the most robust CF-PA biofilms, a 72-h incubation time was chosen. As shown in Fig. 5, 72-h biofilms formed by 12 out of 13 CF-PA strains can be successfully dispersed by S150 within 2 h despite the different response compared with PAO1, with only PA58 being an exception showing tolerance to NO. Microtiter plate results were re-enforced by selected CLSM micrographs in Fig. 6. PAO1 WT, PA10, PA21, PA26, PA30, PA58, and PA68 were selected due to their substantial multilayer biofilm formation in MatTek plates. However, PA58 biofilms formed in MatTek plate also showed significant dispersal (67.5 ± 21% biomass reduction) here. We speculate that the discrepancy was due to the fact that CV staining is a relatively rough method, staining biofilm rings formed at both air-liquid surface and the bottom of each well containing cells and EPS. In contrast, confocal microscopic method measured just total cell mass attached to the bottom of each well. Nevertheless, S150 successfully dispersed biofilms formed by most CF-PA strains tested in this study and thus may also be efficient towards a variety of *P. aeruginosa* strains with different sources and genetic backgrounds.

## Discussion

Low-dose nitric oxide has been repeatedly reported to disperse biofilms formed by different species. Traditionally used SNP was reported to reduce biofilms formed by *P. aeruginosa*, *Vibrio cholerae, Serratia marcescens, Escherichia coli, Bacillus licheniformis* and *Neisseria gonorrhoeae,* with a working concentration ranging from 25 to above 500 nM. (Barraud et al. 2009a; De La Fuente-Núñez et al. 2013; Barnes et al. 2013; Sulemankhil et al. 2012; Falsetta et al. 2009; Jardeleza et al. 2011). A higher concentration of SNP (1–10 μM) was required to disperse biofilms formed by *Fusobacterium nucleatum* and *Staphylococcus epidermidis* (Barraud et al. 2009a)*.* The maximum removal percentage within 24 h ranged from 38.1% in *E. coli* to 92.8% in *B. licheniformis,* and the average removal rate for different species was 63%. Therefore, SNP is effective in dispersing biofilms formed by different bacteria, although each species shows distinct responses. However, when comparing data on SNP-induced *P. aeruginosa* biofilms from various studies, different groups reported using different concentrations of SNP ranging from 500nM to 500 μM (Barraud et al. 2006; Howlin et al. 2011; Barraud et al. 2009a; Chua et al. 2014; Barraud et al. 2009b; Roy et al. 2012). The discrepancy might be due to the fact that SNP does not release NO unless after photolysis or the addition of reducing agents (Kowaluk et al. 1992), resulting in the differences of its efficacy under different laboratory light sources. Furthermore, by-products such as cyanide released from SNP may cause side effects to cells and misinterpretation of experimental results, especially when *P. aeruginosa* is a cyanogenic bacterium (Arnold et al. 1984; Cipollone et al. 2007).

Alternative NO donors with more consistent performances have also been explored. For instance, 200 μM and 1000 μM DETA-NONOate induced the biofilm dispersal of *Shewanella woodyi* and *Staphylococcus aureus*, respectively (Liu et al. 2012; Jardeleza et al. 2011). 50µM and 100μM DPTA NONOate reduced *Shewanella oneidensis* and *Vibrio harveyi* biofilms (Arora et al. 2015; Henares et al. 2013). Direct usage of gaseous NO also led to the dispersal of *Staphylococcus aureus, Acinetobacter baumannii* and *Nitrosomonas europaea* (Sulemankhil et al. 2012; Schmidt et al. 2004). SNAP and GSNO were reported to decrease *P. aeruginosa* biofilms although less effectively than SNP (Barraud et al. 2006), while MAHMA NONOate, PROLI NONOate and Spermine NONOate exhibited higher performance against *P. aeruginosa* biofilms at 20 μM, 40 μM, and 100 μM respectively (Barnes et al. 2013; Barnes et al. 2015; Zhu et al. 2018). However, the half-lives of MAHMA NONOate and PROLI NONOate are 1 min and 1.8 s at 37°*C*, respectively, limiting their applications under many circumstances. In addition to the influences from species and chemical structure of the donors that contribute substantially to the releasing efficiency, other environmental conditions such as temperature, pH and metal ion also play a role in the efficiency of NO donors with different releasing mechanisms. For instance, the addition of Cu^2+^ to reaction solutions can accelerate the NO release of SNP, GSNO and SNAP (Smith and Dasgupta 2002; Megson et al. 1999). Basic environment (pH above 8) can decrease the release rates of S-nitrosothiols and NONOates (Hornyák et al. 2012; Li et al. 2020). Taken all these factors into consideration, in this study, we tested 7 NO donors from different categories, including SNP, SNAP, GSNO, PROLI NONOate, MAHMA NONOate (NOC-9), DEA-NONOate and Spermine NONOate (S150), with different concentrations and exposure times under the same condition. Figure 1 showed that 24-h 250 μM SNP treatment and 2-h 250 μM S150 treatment can effectively disperse *P. aeruginosa* PAO1 WT biofilms in microtiter plates to ~ 60%. The optimal concentration of SNP (250 μM–500 μM) from this study is not consistent with the nanomolar range (Barraud et al. 2006 and 2009a) but agreed with Roy et al. 2012 and Howlin et al. 2017. The required time for SNP treatment for at least 30% biomass reduction, i.e. at least 12 h, is consistent with Barnes et al. 2013. As such, our study further demonstrated that SNP can efficiently disperse biofilms, but the optimal concentration varies greatly under different settings. Interestingly, while our data did not show a significant role of MAHMA NONOate and PROLI NONOate (Supplementary S3 and S4) as reported in some earlier studies from Barnes et al. in 2013 and 2015 comparing different NO donors, our selection of Spermine NONOate is in accordance with the latest NO donor used by the same group (Zhu et al. 2018). Although the reported concentration and treatment time for Spermine NONOate was 100 μM and 15 mins, leading to 88% biofilm reduction, the assay was conducted on very early stage PAO1 biofilms (~ 6 h). Hence, the optimal dosage of Spermine NONOate determined on 24-h PAO1 biofilms in this study, i.e. a higher concentration (250 μM), longer treatment time (2 h) and slightly lower biofilm removal rate (~ 60%), is reasonable and can be regarded as in accordance with the previous study.

Gas phase chemiluminescence (CLD) results have shown that while the same molarity of SNP is expected to release 50% of NO less than S150, it actually released 78.7% less NO within 1.5 h **(**Fig. 2a and b**)**. Furthermore, the decomposition of S150 started as soon as the compound was in contact with the solution, corresponding to its spontaneous NO release following the first-order kinetics (Ramamurthi and Lewis 1997). In contrast, SNP remained a constant but slow release state under normal room lighting. The lower efficacy of SNP was further confirmed by results in Fig. 2c, where SNP can only generate NO in the presence of intense light. Even so, 500 μM SNP only reached the maximum 0.8 ppm NO release peak compared with 0.35 ppm peak from 5 μM S150. As S150 can efficiently release NO with or without light, it is more suitable for different applications such as in patients.

While it was shown that S150 efficiently releases NO, Figs. 3 and 4 confirmed that the biofilm dispersal effect came from NO rather than any by-product or side effect. When NO scavenger was added, biofilm dispersal was abolished. Neither SNP nor S150 posed toxic effects towards planktonic cells, indicating the biofilm removal was not due to cytotoxicity. Interestingly, both NO donors significantly enhanced bacteria numbers without additional nutrients added into M9 media. As NO radical can be readily converted to nitrite and nitrate, and previous studies have shown that nitrate can support the growth of *P. aeruginosa* in anaerobic/anoxic environment (Fang et al. 2013; Yoon et al. 2011; Line et al. 2014), the additional nitrite or nitrate might have contributed to the increased growth of planktonic *P. aeruginosa* cells. Alternatively, as the backbone of S150 is rich in carbon and nitrogen, while cyanide can be degraded and utilized as nutrients (Cipollone et al. 2007; Knowles 1988), the chemical breakdown products from SNP and S150 may have acted as additional nutrients for cell growth during the treatment. However, the precise mechanism of S150/SNP-induced growth increase is yet to be elucidated. Some previous studies used ‘increased CFU/OD/turbidity in the effluents’ after SNP treatment as the indicator of biofilm dispersal into planktonic forms (Barraud et al. 2006; Howlin et al. 2017; Chua et al. 2014). Depending on the treatment period, this measurement may not be accurate enough to reflect the dispersal rate, as the donors may have enhanced the planktonic growth at the same time. In summary, 250 μM S150 is the optimal NO donor dosage among all compounds tested here for triggering *P. aeruginosa* biofilms. Additionally, due to its desirable decomposition half-life (39 mins at pH 7.4, 37°C) compared with other short half-life NONOates such as MAHMA NONOates and PROLI NONOates, it is easier to prepare and control under different conditions.

Darling and Evans (2003) reported that NO production in vivo reduced *P. aeruginosa* adherence to human bronchial epithelial cells and enhanced the killing of internalized bacteria. However, various publications indicated that exhaled NO from CF patients is reduced compared with that produced by normal patients (Elphick et al. 1999; Jöbsis et al. 2000; Mhanna et al. 2001). In healthy individuals, inducible NOS (iNOS) is expressed maximally following an inflammatory stimulus and produces large, micromolar scale of NO (Darling and Evans 2003). However, in CF patients’ airways with chronic severe inflammation, the amount of exhaled NO is not increased and the expression of epithelial iNOS is reduced (Darling and Evans 2003). Therefore, CF-PA biofilms developed in chronic lung infection may be constantly exposed to sublethal dosage of NO. A recent study stated that pre-treated biofilms with non-dispersing concentrations of NO showed much increased tolerance to NO (Zhu et al. 2018). As CF-PAs usually exhibit much genetic variation compared with type strains due to in vivo adaption, the repeated NO exposure may lead to mutations resulting in higher tolerance to NO. Therefore, we suspected that the biofilms formed by CF-PAs isolated from sputum samples could develop tolerant to NO. From Figs. 5 and 6**,** it can be concluded that biofilms formed by different CF-PAs sampled from non-familiar patients were successfully dispersed by S150, regardless of their total biomass. Our data suggested that S150 can potentially prevent biofilm formation or disperse pre-established biofilms in clinical settings by direct application or incorporation into medical devices such as bone cement, dermal fillers and wound dressing. It may also be applied in combination with antibiotics to increase the susceptibility of cells encased in biofilms.

## Electronic supplementary material

ESM 1 (DOCX 10894 kb)

## References

[CR1] Aaron SD, Kottachchi D, Ferris WJ, Vandemheen KL, St Denis ML, Plouffe A, Doucette AP, Saginur R, Chan FT, Ramotar K (2004). Sputum versus bronchoscopy for diagnosis of *Pseudomonas aeruginosa* biofilms in cystic fibrosis. Eur Respir J.

[CR2] Arnold WP, Longnecker DE, Epstein RM (1984). Photodegradation of sodium nitroprusside: biologic activity and cyanide release. Anesthesiology.

[CR3] Arora DP, Hossain S, Xu Y, Boon EM (2015). Nitric oxide regulation of bacterial biofilms. Biochemistry..

[CR4] Barnes RJ, Bandi RR, Wong WS, Barraud N, McDougald D, Fane A, Kjelleberg S, Rice SA (2013). Optimal dosing regimen of nitric oxide donor compounds for the reduction of *Pseudomonas aeruginosa* biofilm and isolates from wastewater membranes. Biofouling.

[CR5] Barnes RJ, Low JH, Bandi RR, Tay M, Chua F, Aung T, Fane AG, Kjelleberg S, Rice SA (2015). Nitric oxide treatment for the control of reverse osmosis membrane biofouling. Appl Environ Microbiol.

[CR6] Barraud N, Hassett DJ, Hwang SH, Rice SA, Kjelleberg S, Webb JS (2006). Involvement of nitric oxide in biofilm dispersal of *Pseudomonas aeruginosa*. J Bacteriol.

[CR7] Barraud N, Storey MV, Moore ZP, Webb JS, Rice SA, Kjelleberg S (2009). Nitric oxide-mediated dispersal in single- and multi-species biofilms of clinically and industrially relevant microorganisms. Microb Biotechnol.

[CR8] Barraud N, Schleheck D, Klebensberger J, Webb JS, Hassett DJ, Rice SA, Kjelleberg S (2009). Nitric oxide signaling in *Pseudomonas aeruginosa* biofilms mediates phosphodiesterase activity, decreased cyclic di-GMP levels, and enhanced dispersal. J Bacteriol.

[CR9] Belcastro E, Wu W, Fries-Raeth I, Corti A, Pompella A, Leroy P, Lartaud I, Gaucher C (2017). Oxidative stress enhances and modulates protein S-nitrosation in smooth muscle cells exposed to S- nitrosoglutathione. Nitric Oxide.

[CR10] Bianconi I, D'Arcangelo S, Esposito A, Benedet M, Piffer E, Dinnella G, Gualdi P, Schinella M, Baldo E, Donati C, Jousson O (2019). Persistence and microevolution of *Pseudomonas aeruginosa* in the cystic fibrosis lung: a single-patient longitudinal genomic study. Front Microbiol.

[CR11] Blau H, Linnane B, Carzino R, Tannenbaum EL, Skoric B, Robinson PJ, Robertson C, Ranganathan SC (2014). Induced sputum compared to bronchoalveolar lavage in young, non-expectorating cystic fibrosis children. J Cyst Fibros.

[CR12] Brisbois EJ, Hand H, Major TC, Bartlett RH, Meyerhoff ME (2013). Long-term nitric oxide release and elevated temperature stability with S-Nitroso-N-acetylpenicillamine (SNAP)-doped elast-eon E2As polymer. Biomaterials..

[CR13] Caçador NC, Capizzani CPDC, Torres LAGMM, Galetti R, Ciofu O, Darini ALDC, Høiby N (2018). Adaptation of *Pseudomonas aeruginosa* to the chronic phenotype by mutations in the algTmucABD operon in isolates from Brazilian cystic fibrosis patients. PLoS One.

[CR14] Cathie K, Howlin RP, Carroll M, Clarke S, Connett G, Cornelius V, Daniels T, Duignan C, Hall-Stoodley L, Jefferies J, Kelso M, Kjelleberg S, Legg J, Pink S, Rogers G, Salib R, Stoodley P, Sukhtankar P, Webb JS, Faust SN (2014). G385 RATNO - reducing antibiotic tolerance using nitric oxide in cystic fibrosis: report of a proof of concept clinical trial. Arch Dis Child.

[CR15] Chua SL, Liu Y, Yam JKH, Chen Y, Vejborg RM, Tan BGC, Kjelleberg S, Tolker-Nielsen T, Givskov M, Yang L (2014). Dispersed cells represent a distinct stage in the transition from bacterial biofilm to planktonic lifestyles. Nat Commun.

[CR16] Ciofu O, Tolker-Nielsen T (2019) tolerance and resistance of *Pseudomonas aeruginosa* biofilms to antimicrobial agents—how P. aeruginosa can escape antibiotics. Front Microbial 10:913 10.3389/fmicb.2019.00913 PMC6509751 31130925

[CR17] Cipollone R, Frangipani E, Tiburzi F, Imperi F, Ascenzi P, Visca P (2007). Involvement of *Pseudomonas aeruginosa* rhodanese in protection from cyanide toxicity. Appl Environ Microbiol.

[CR18] Darling KE, Evans TJ (2003) Effects of nitric oxide on *Pseudomonas aeruginosa* infection of epithelial cells from a human respiratory cell line derived from a patient with cystic fibrosis. Infect Immun 71:2341–2349 10.1128/IAI.71.5.2341-2349.2003 PMC153226 12704103

[CR19] De La Fuente-Núñez C, Reffuveille F, Fairfull-Smith KE, Hancock REW (2013). Effect of nitroxides on swarming motility and biofilm formation, multicellular behaviors in *Pseudomonas aeruginosa*. Antimicrob Agents Chemother.

[CR20] De Vos D, Jr Lim A, Pirnay JP, Struelens M, Vandenvelde C, Duinslaeger L, Vanderkelen A, Cornelis P (1997). Direct detection and identification of *Pseudomonas aeruginosa* in clinical samples such as skin biopsy specimens and expectorations by multiplex PCR based on two outer membrane lipoprotein genes, oprI and oprL. J Clin Microbiol.

[CR21] Duong HTT, Jung K, Kutty SK, Agustina S, Adnan NNM, Basuki JS, Kumar N, Davis TP, Barraud N, Boyer C (2014). Nanoparticle (star polymer) delivery of nitric oxide effectively negates *Pseudomonas aeruginosa* biofilm formation. Biomacromolecules.

[CR22] Duong HTT, Adnan NNM, Barraud N, Basuki JS, Kutty SK, Jung K, Kumar N, Davis TP, Boyer C (2014). Functional gold nanoparticles for the storage and controlled release of nitric oxide: applications in biofilm dispersal and intracellular delivery. J Mater Chem B.

[CR23] Elphick HE, Demoncheaux E, Ritson S, Higenbottam TW, Everard ML (1999). Exhaled nitric oxide is decreased in infants with cystic fibrosis. Thorax.

[CR24] Falsetta ML, Bair TB, Ku SC, vanden Hoven RN, Steichen CT, McEwan AG, Jennings MP, Apicella MA (2009). Transcriptional profiling identifies the metabolic phenotype of gonococcal biofilms. Infect Immun.

[CR25] Fang H, Toyofuku M, Kiyokawa T, Ichihashi A, Tateda K, Nomura N (2013). The impact of anaerobiosis on strain-dependent phenotypic variations in *Pseudomonas aeruginosa*. Biosci Biotechnol Biochem.

[CR26] Gilligan PH (1991). Microbiology of airway disease in patients with cystic fibrosis. Clin.Microbiol.Rev..

[CR27] Govan JRW, Deretic V (1996). Microbial pathogenesis in cystic fibrosis: mucoid *Pseudomonas aeruginosa* and *Burkholderia cepacia*. Microbiol Rev.

[CR28] Henares MB, Xu Y, Boon ME (2013). A nitric oxide-responsive quorum sensing circuit in *Vibrio harveyi* regulates flagella production and biofilm formation. Int J Mol Sci.

[CR29] Heydorn A, Nielsen AT, Hentzer M, Sternberg C, Givskov M, Ersbøll BK, Molin S (2000). Quantification of biofilm structures by the novel computer program COMSTAT. Microbiology.

[CR30] Høiby N, Frederiksen B, Pressler T (2005). Eradication of early *Pseudomonas aeruginosa* infection. J Cyst Fibros.

[CR31] Hornyák I, Marosi K, Kiss L, Gróf P, Lacza Z (2012). Increased stability of S-Nitrosothiol solutions via pH modulations. Free Radic Res.

[CR32] Howlin R, Cathie K, Hall-Stoodley L, Niehaus L, Connett G, Legg G, Daniels T, Carroll M, Jefferies J, Clarke SC, Stoodley P, Webb JS, Faust SN (2011). Nitric oxide-mediated dispersal and enhanced antibiotic sensitivity in *Pseudomonas aeruginosa* biofilms from the cystic fibrosis lung. Arch Dis Child.

[CR33] Howlin RP, Cathie K, Hall-Stoodley L, Cornelius V, Duignan C, Allan RN, Fernandez BO, Barraud N, Bruce KD, Jefferies J, Kelso M, Kjelleberg S, Rice SA, Rogers GB, Pink S, Smith C, Sukhtankar PS, Salib R, Legg J, Carroll M, Daniels T, Feelisch M, Stoodley P, Clarke SC, Connett G, Faust SN, Webb JS (2017). Low-dose nitric oxide as targeted anti-biofilm adjunctive therapy to treat chronic *Pseudomonas aeruginosa* infection in cystic fibrosis. Mol Ther.

[CR34] Jardeleza C, Foreman A, Baker L, Paramasivan S, Field J, Tan LW, Wormald PJ (2011). The effects of nitric oxide on *Staphylococcus aureus* biofilm growth and its implications in chronic rhinosinusitis. Int Forum Allergy Rhinol.

[CR35] Jöbsis Q, Raatgeep HC, Schellekens SL, Kroesbergen A, Hop WC, de Jongste JC (2000). Hydrogen peroxide and nitric oxide in exhaled air of children with cystic fibrosis during antibiotic treatment. Eur Respir J.

[CR36] Knowles CJ (1988). Cyanide utilization and degradation by microorganisms. CIBA Found Symp.

[CR37] Kowaluk E, Seth P, Fung HL (1992). Metabolic activation of sodium nitroprusside to nitric oxide in vascular smooth muscle. J Pharmacol Exp Ther.

[CR38] Li B, Ming Y, Liu Y, Xing H, Fu R, Li Z, Ni R, Li L, Duan D, Xu J, Li C, Xiang M, Song H, Chen J (2020). Recent developments in pharmacological effect, mechanism and application prospect of diazeniumdiolates. Front Pharmacol.

[CR39] Line L, Alhede M, Kolpen M, Kühl M, Ciofu O, Bjarnsholt T, Moser C, Toyofuku M, Nomura N, Høiby N, Jensen PØ (2014). Physiological levels of nitrate support anoxic growth by denitrification of *Pseudomonas aeruginosa* at growth rates reported in cystic fibrosis lungs and sputum. Front Microbiol.

[CR40] Liu N, Xu Y, Hossain S, Huang N, Coursolle D, Gralnick JA, Boon EM (2012). Nitric oxide regulation of cyclic di-GMP synthesis and hydrolysis in *Shewanella woodyi*. Biochemistry..

[CR41] Maragos CM, Morley D, Wink DA, Dunams TM, Saavedra JE, Hoffman A, Bove AA, Isaac L, Hrabie JA, Keefer LK (1991). Complexes of .NO with nucleophiles as agents for the controlled biological release of nitric oxide. Vasorelaxant effects. J Med Chem.

[CR42] Marvasi M, Chen C, Carrazana M, Durie IA, Teplitski M (2014). Systematic analysis of the ability of nitric oxide donors to dislodge biofilms formed by *Salmonella enterica* and *Escherichia coli* O157:H7. AMB Express.

[CR43] Megson IL, Morton S, Greig IR, Mazzei FA, Field RA, Butler AR, Caron G, Gasco A, Fruttero R, Webb DJ (1999). N-substituted analogues of S-nitroso-n-acetyl-d,l-penicillamine: chemical stability and prolonged nitric oxide mediated vasodilatation in isolated rat femoral arteries. Br J Pharmacol.

[CR44] Mena M, Smith EE, Burns JL, Speert DP, Moskowitz SM, Perez JL, Oliver A (2008). Genetic adaptation of *Pseudomonas aeruginosa* to the airways of cystic fibrosis patients is catalyzed by hypermutation. J Bacteriol.

[CR45] Mhanna MJ, Ferkol T, Martin RJ, Dreshaj IA, van Heeckeren AM, Kelley TJ, Haxhiu MA (2001). Nitric oxide deficiency contributes to impairment of airway relaxation in cystic fibrosis mice. Am J Respir Cell Mol Biol.

[CR46] Miles AA, Misra SS, Irwin JO (1938). The estimation of the bactericidal power of the blood. Epidemiol Infect.

[CR47] Mulcahy LR, Burns JL, Lory S, Lewis K (2010). Emergence of *Pseudomonas aeruginosa* strains producing high levels of persister cells in patients with cystic fibrosis. J Bacteriol.

[CR48] Nablo BJ, Schoenfisch MH (2003). Antibacterial properties of nitric oxide-releasing sol-gels. J Biomed Mater Res Part A.

[CR49] Nablo BJ, Rothrock AR, Schoenfisch MH (2005). Nitric oxide-releasing sol-gels as antibacterial coatings for orthopedic implants. Biomaterials.

[CR50] Napoli C, Ignarro LJ (2003). Nitric oxide-releasing drugs. Annu Rev Pharmacol Toxicol.

[CR51] Piknova B, Schechter AN (2011). Measurement of nitrite in blood samples using the ferricyanide-based hemoglobin oxidation assay. Methods Mol Biol.

[CR52] Ramamurthi A, Lewis RS (1997). Measurement and modeling of nitric oxide release rates for nitric oxide donors. Chem Res Toxicol.

[CR53] Rinaldo S, Giardina G, Mantoni F, Paone A, Cutruzzolà F (2018) Beyond nitrogen metabolism: nitric oxide, cyclic-di-GMP and bacterial biofilms. FEMS Microbiol Lett. 10.1093/femsle/fny029 10.1093/femsle/fny029 29401255

[CR54] Römling U, Galperin MY, Gomelsky M (2013). Cyclic di-GMP: the first 25 years of a universal bacterial second messenger. Microbiol Mol Biol Rev.

[CR55] Roy AB, Petrova OE, Sauer K (2012). The phosphodiesterase DipA (PA5017) is essential for *Pseudomonas aeruginosa* biofilm dispersion. J Bacteriol.

[CR56] Sadrearhami Z, Yeow J, Nguyen TK, Ho KKK, Kumar N, Boyer C (2017). Biofilm dispersal using nitric oxide loaded nanoparticles fabricated by photo-PISA: influence of morphology. Chem Commun.

[CR57] Saiman L, Mehar F, Niu WW, Neu HC, Shaw KJ, Miller G, Prince A (1996). Antibiotic susceptibility of multiple resistant *Pseudomonas aeruginosa* isolated from patients with cystic fibrosis, including candidates for transplantation. Clin.Inf.Dis..

[CR58] Schmidt I, Steenbakkers PJM, op den Camp HJM, Schmidt K, Jetten MSM (2004). Physiologic and proteomic evidence for a role of nitric oxide in biofilm formation by *Nitrosomonas europaea* and other ammonia oxidizers. J Bacteriol.

[CR59] Shen Z, He K, Ding Z, Zhang M, Yu Y, Hu J (2019). Visible-light-triggered self-reporting release of nitric oxide (NO) for bacterial biofilm dispersal. Macromolecules.

[CR60] Smith JN, Dasgupta TP (2002). Mechanism of nitric oxide release. I Two-electron reduction of sodium nitroprusside by l-cysteine in aqueous solution. Inorg React Mech.

[CR61] Soren O, Rineh A, Silva D, Cai Y, Howlin RP, Allan RN, Feelisch M, Davies JC, Connett GJ, Faust SN, Kelso MJ, Webb JS (2019). Cephalosporin nitric oxide-donor prodrug DEA-C3D disperses biofilms formed by clinical cystic fibrosis isolates of *Pseudomonas aeruginosa*. J Antimicrob Chemother.

[CR62] Sulemankhil I, Ganopolsky JG, Dieni CA, Dan AF, Jones ML, Prakash S (2012). Prevention and treatment of virulent bacterial biofilms with an enzymatic nitric oxide-releasing dressing. Antimicrob Agents Chemother.

[CR63] Winstanley C, O’Brien S, Brockhurst MA (2016). *Pseudomonas aeruginosa* evolutionary adaptation and diversification in cystic fibrosis chronic lung infections. Trends Microbiol.

[CR64] Yang T, Zelikin AN, Chandrawati R (2018). Progress and promise of nitric oxide-releasing platforms. Adv Sci (Weinh).

[CR65] Yoon MY, Lee KM, Park Y, Yoon SS (2011). Contribution of cell elongation to the biofilm formation of *Pseudomonas aeruginosa* during anaerobic respiration. PLoS One.

[CR66] Zhu X, Oh HS, Ng YCB, Tang PY, Barraud N, Rice SA (2018). Nitric oxide-mediated induction of dispersal in *Pseudomonas aeruginosa* biofilms is inhibited by flavohemoglobin production and is enhanced by imidazole. Antimicrob Agents Chemother.

