# Supplementary material for: Optimization of nitric oxide donors for investigating biofilm dispersal response in Pseudomonas aeruginosa clinical isolates
Source: Appl Microbiol Biotechnol. 2020 Aug 31;104(20):8859–69. doi: 10.1007/s00253-020-10859-7 (PMC7502453; doi:10.1007/s00253-020-10859-7)

**Supplement to *Applied Microbiology and Biotechnology***

**Optimization of nitric oxide donors for investigating biofilm dispersal response in *Pseudomonas aeruginosa* clinical isolates**

Yu-ming Cai^a*^, Jeremy S. Webb^a^

^a^ National Biofilms Innovation Centre, University of Southampton, Southampton, SO17 1BJ, UK

Corresponding author: Dr. Yu-ming Cai, E-mail: [yc2m18@soton.ac.uk](mailto:yc2m18@soton.ac.uk)

**Supplementary Table S1:** Bacterial strains used in this study

| **Strains and plasmids** | **Genotype or phenotypes^a^** | Reference |
| --- | --- | --- |
|  |  |  |
| PAO1 | Wild-Type, C.Manoil lab, University of Washington |  |
|  | | |
|  | | |
| **Clinical *P. aeruginosa* strains isolated from cystic fibrosis patients** | | |
| PA10 | *P. aeruginosa* clinical isolates from Southampton General hospital CF patients | Sputum samples from 72 patients with CF (median age at informed consent 21 years, range 17-62; UK NHS Research Ethics Reference 08/H0502/126 |
| PA15 | *P. aeruginosa* clinical isolates from Southampton General hospital CF patients |  |
| PA20 | *P. aeruginosa* clinical isolates from Southampton General hospital CF patients |  |
| PA21 | *P. aeruginosa* clinical isolates from Southampton General hospital CF patients |  |
| PA26 | *P. aeruginosa* clinical isolates from Southampton General hospital CF patients |  |
| PA30 | *P. aeruginosa* clinical isolates from Southampton General hospital CF patients |  |
| PA37 | *P. aeruginosa* clinical isolates from Southampton General hospital CF patients |  |
| PA39 | *P. aeruginosa* clinical isolates from Southampton General hospital CF patients |  |
| PA44 | *P. aeruginosa* clinical isolates from Southampton General hospital CF patients |  |
| PA49 | *P. aeruginosa* clinical isolates from Southampton General hospital CF patients |  |
| PA55 | *P. aeruginosa* clinical isolates from Southampton General hospital CF patients |  |
| PA56 | *P. aeruginosa* clinical isolates from Southampton General hospital CF patients |  |
| PA57 | *P. aeruginosa* clinical isolates from Southampton General hospital CF patients |  |
| PA58 | *P. aeruginosa* clinical isolates from Southampton General hospital CF patients |  |
| PA66 | *P. aeruginosa* clinical isolates from Southampton General hospital CF patients |  |
|  |  |  |

**Figure S1.** NaNO_2_ standard curves for NO release calibration (a) NO gas standards using 250 pmol, 375 pmol, 500 pmol, 750 pmol and 1000 pmol NaNO_2_. (b) Standard curve generated using NO gas amount as Y axis and areas under the peak as X axis. 1 pmol NaNO_2_=1 pmol NO. n=3 independent experiments.

**Figure S2.** *P. aeruginosa* PAO1 WT biofilms treated with SNP for (a) 1 hr, (b) 2 hrs, (c) 4 hrs, (d) 6 hrs, (e) 8 hrs, (f) 12 hrs and (g) 24 hrs. ** denotes 0.01<P<0.05, *** denotes P<0.01. n=3 independent experiments × 6 technical replicates

**Figure S3.** *P. aeruginosa* PAO1 WT biofilms treated with MAHMA NONOate for (a) 1 hr, (b) 2 hrs, (c) 4 hrs, (d) 6 hrs, (e) 8 hrs, (f) 12 hrs and (g) 24 hrs. ** denotes 0.01<P<0.05, *** denotes P<0.01. n=3 independent experiments × 6 technical replicates

**Figure S4.** *P. aeruginosa* PAO1 WT biofilms treated with PROLI NONOate for (a) 1 hr, (b) 2 hrs, (c) 4 hrs, (d) 6 hrs, (e) 8 hrs, (f) 12 hrs and (g) 24 hrs. ** denotes 0.01<P<0.05, *** denotes P<0.01. n=3 independent experiments × 6 technical replicates

**Figure S5.** *P. aeruginosa* PAO1 WT biofilms treated with DEA NONOate for (a) 1 hr, (b) 2 hrs, (c) 4 hrs, (d) 6 hrs, (e) 8 hrs, (f) 12 hrs and (g) 24 hrs. ** denotes 0.01<P<0.05, *** denotes P<0.01. n=3 independent experiments × 6 technical replicates

**Figure S6.** *P. aeruginosa* PAO1 WT biofilms treated with GSNO for (a) 1 hr, (b) 2 hrs, (c) 4 hrs, (d) 6 hrs, (e) 8 hrs, (f) 12 hrs and (g) 24 hrs. ** denotes 0.01<P<0.05, *** denotes P<0.01. n=3 independent experiments × 6 technical replicates

**Figure S7.** *P. aeruginosa* PAO1 WT biofilms treated with S150 for (a) 1 hr, (b) 2 hrs, (c) 4 hrs, (d) 6 hrs, (e) 8 hrs, (f) 12 hrs and (g) 24 hrs. ** denotes 0.01<P<0.05, *** denotes P<0.01. n=3 independent experiments × 6 technical replicates

**Figure S8.** *P. aeruginosa* PAO1 WT biofilms treated with SNAP for (a) 1 hr, (b) 2 hrs, (c) 4 hrs, (d) 6 hrs, (e) 8 hrs, (f) 12 hrs and (g) 24 hrs. ** denotes 0.01<P<0.05, *** denotes P<0.01. n=3 independent experiments × 6 technical replicates


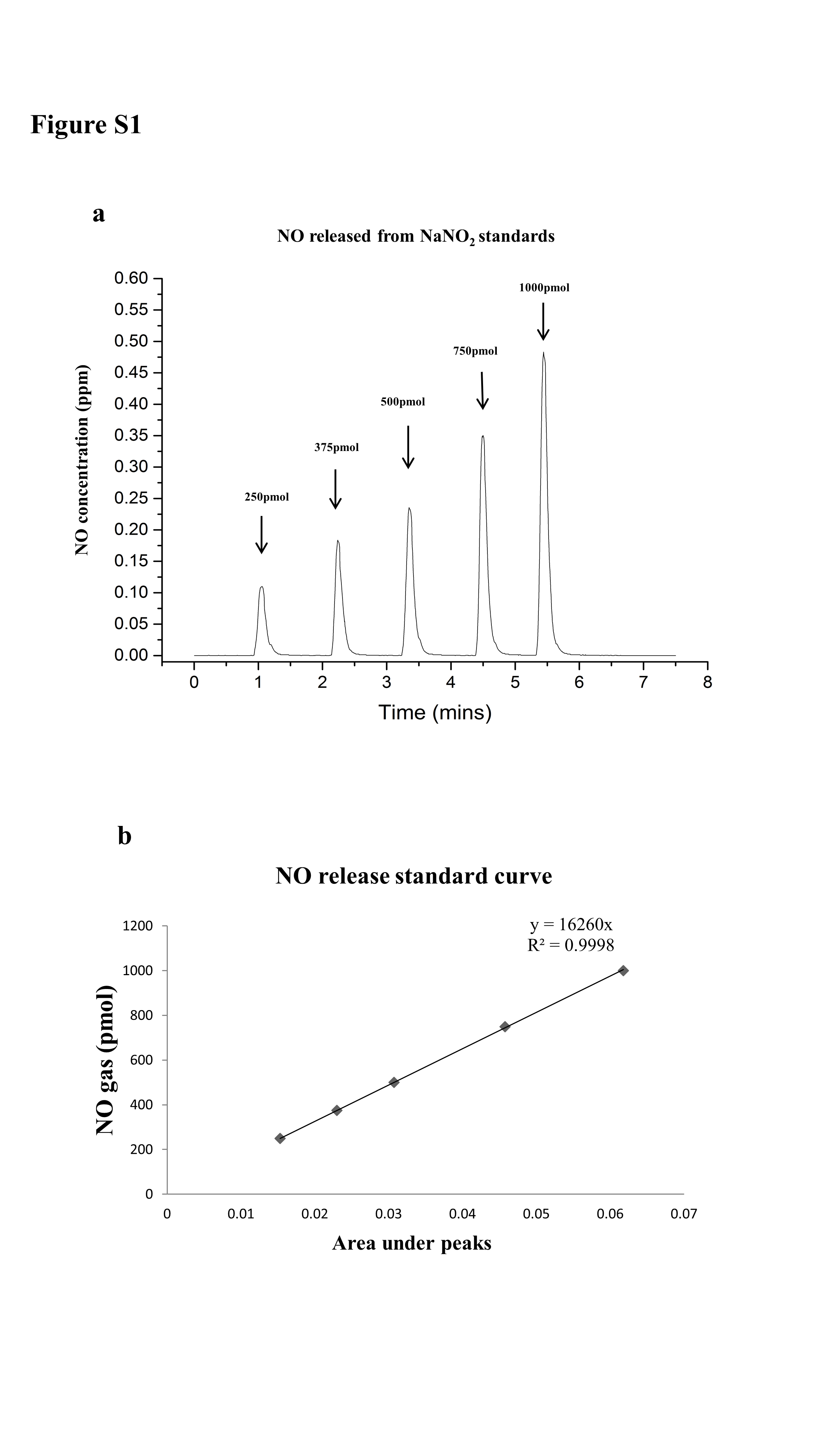


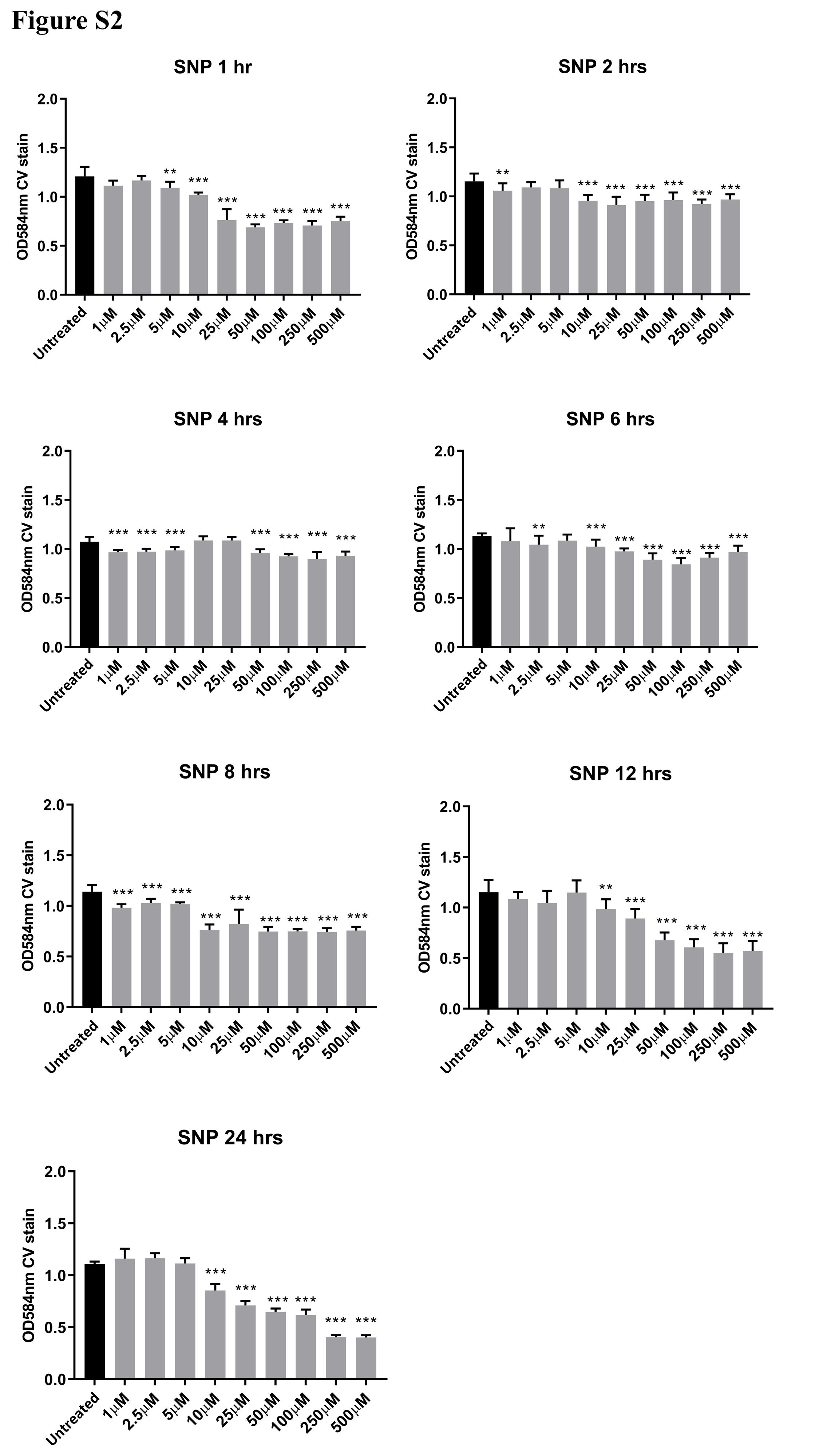


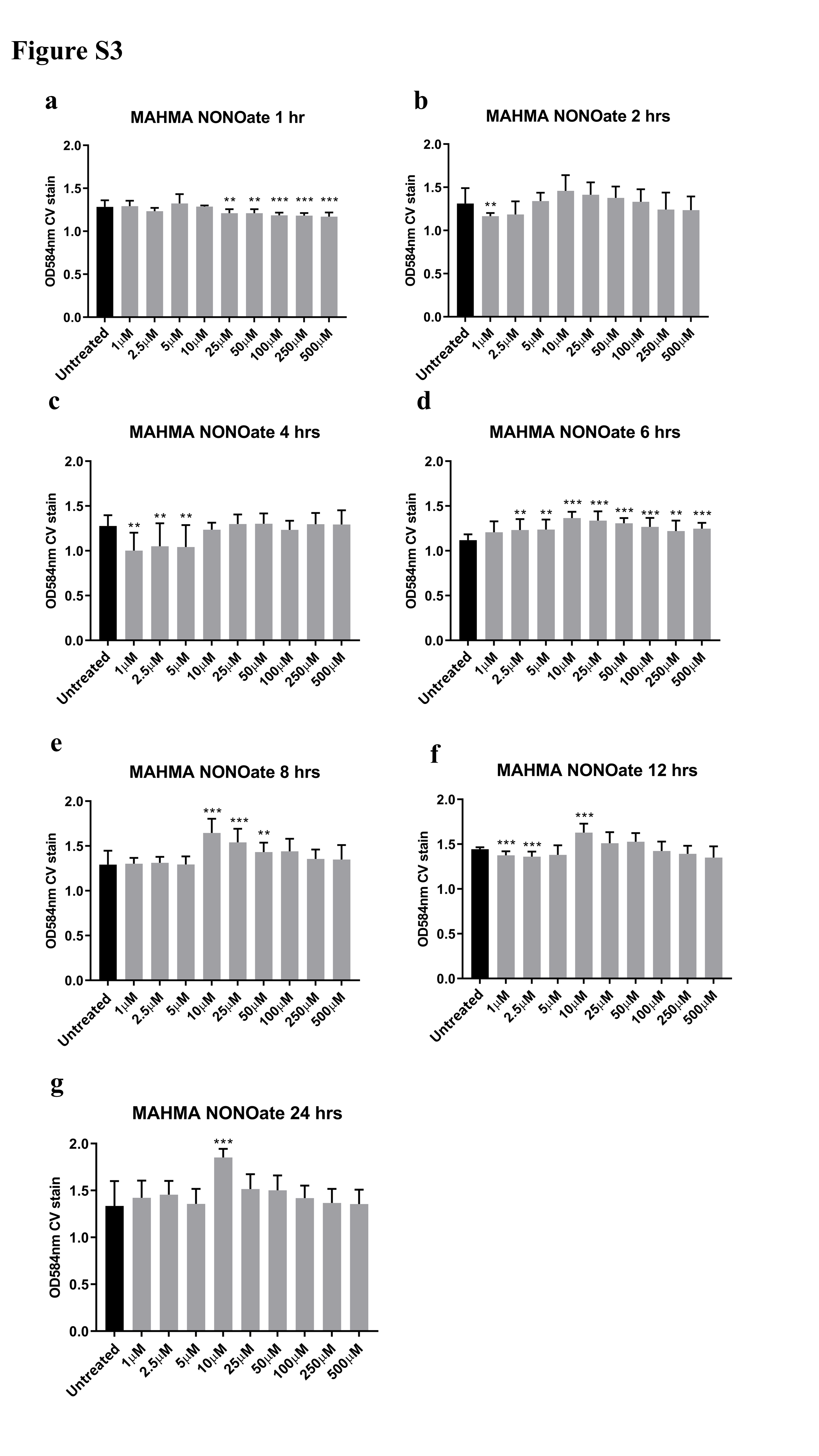


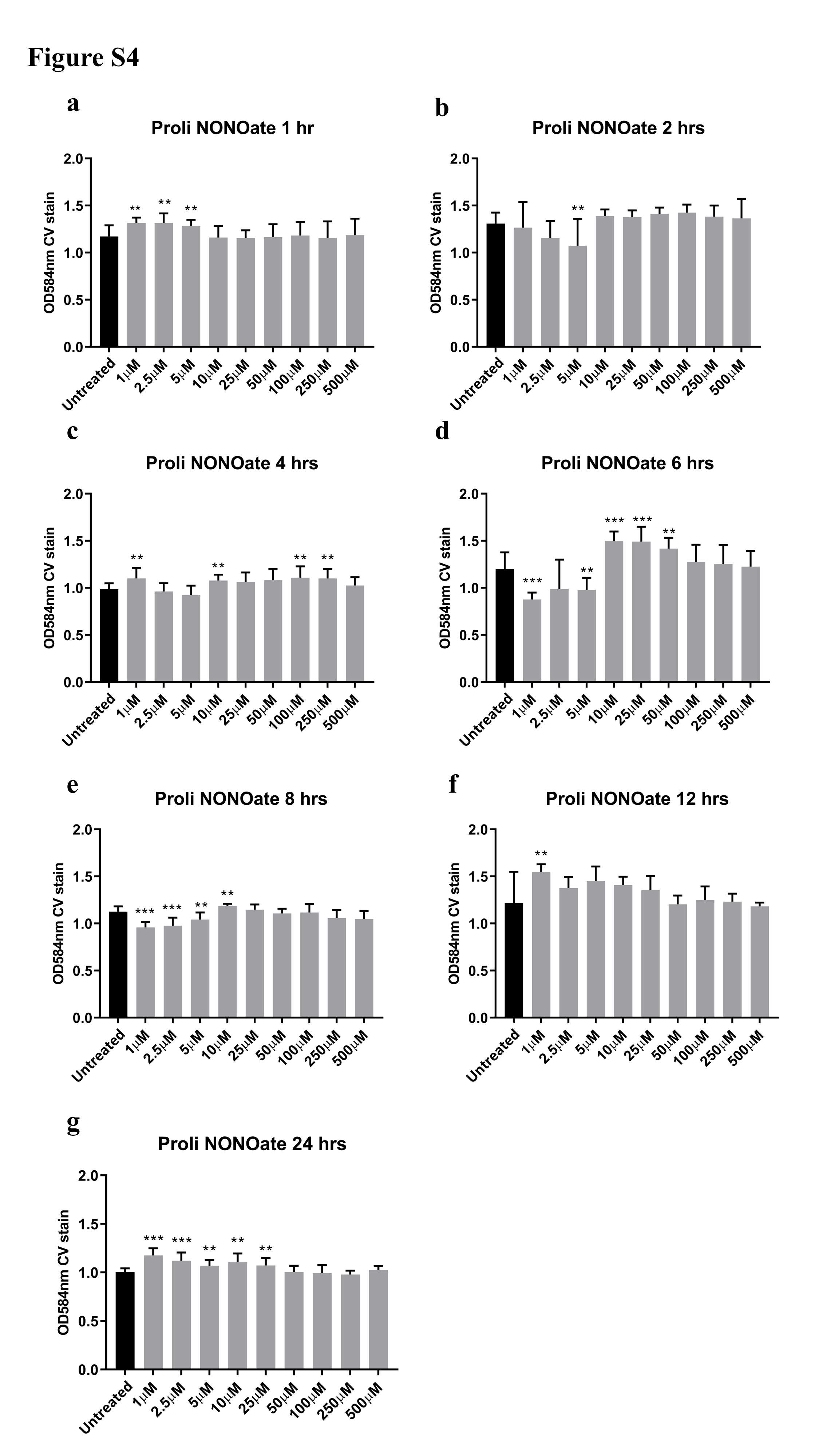


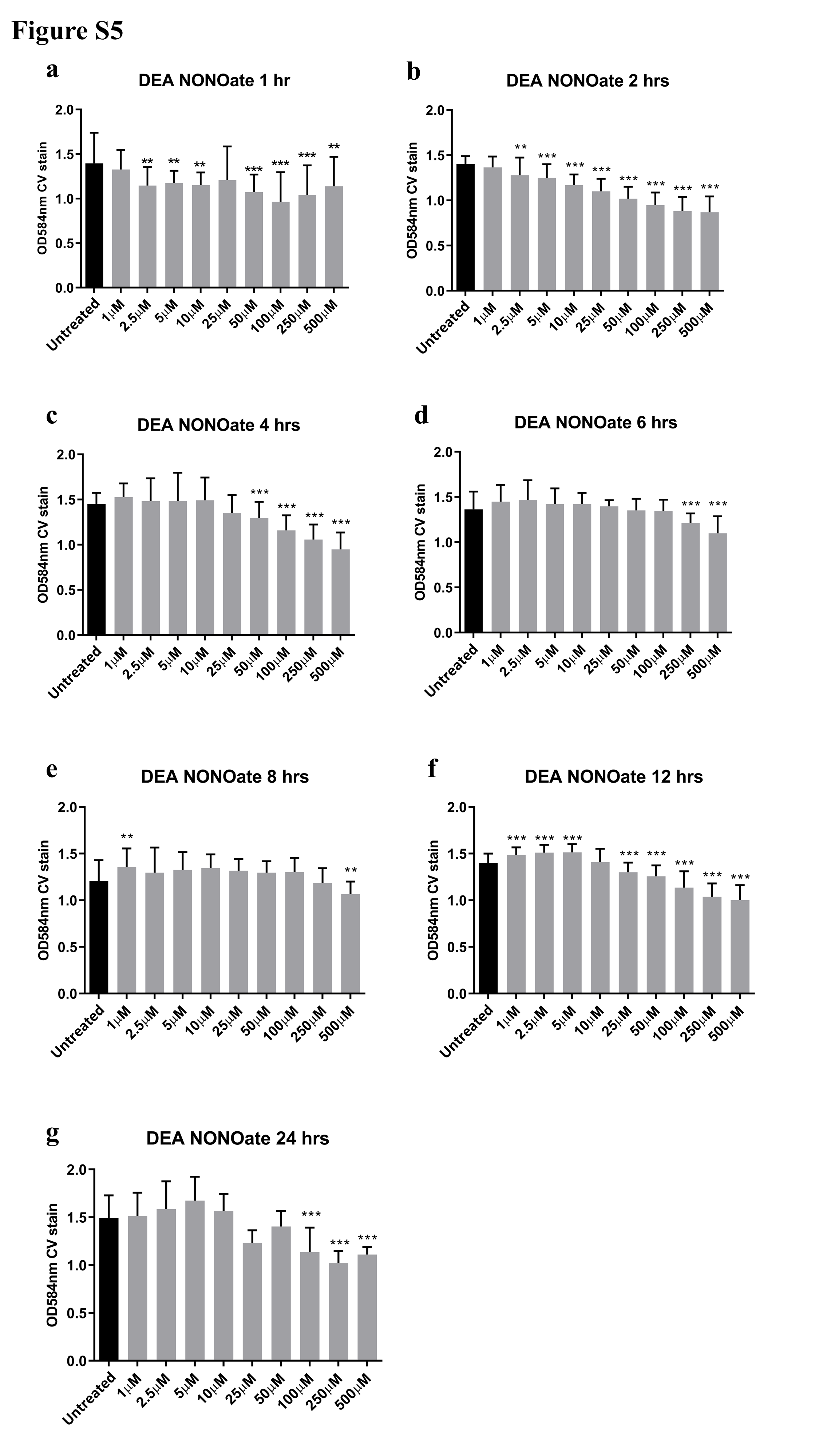


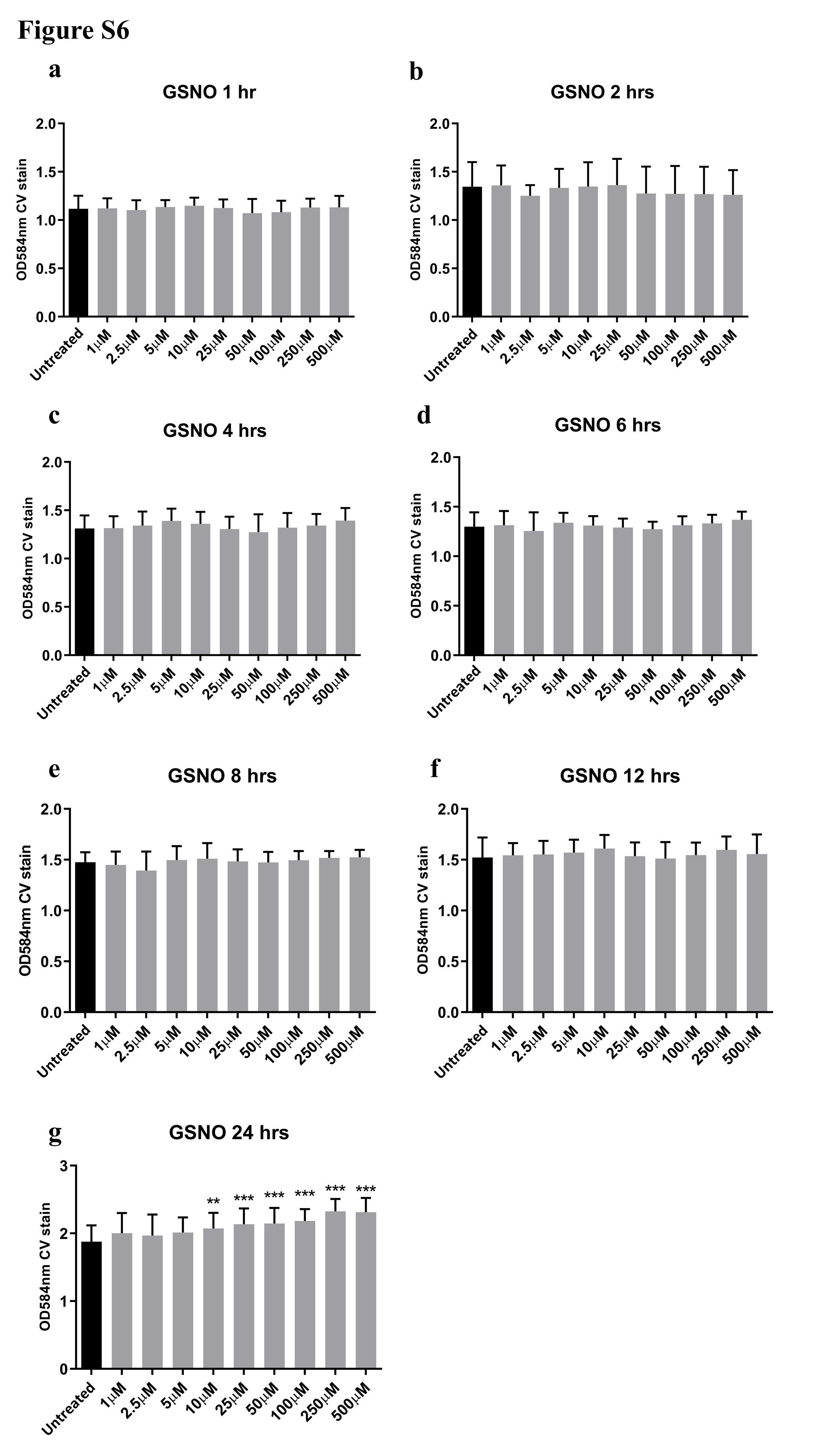


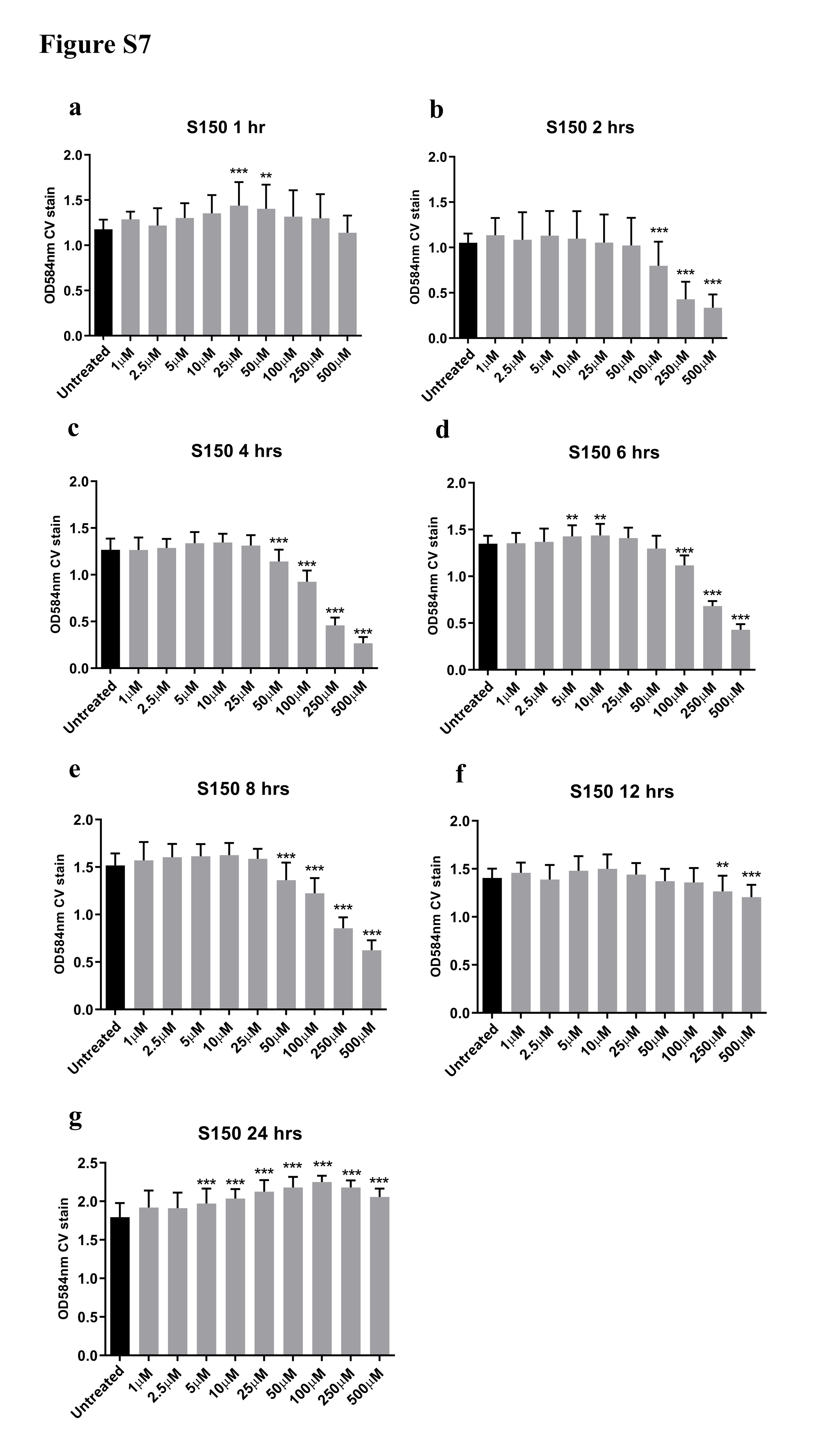


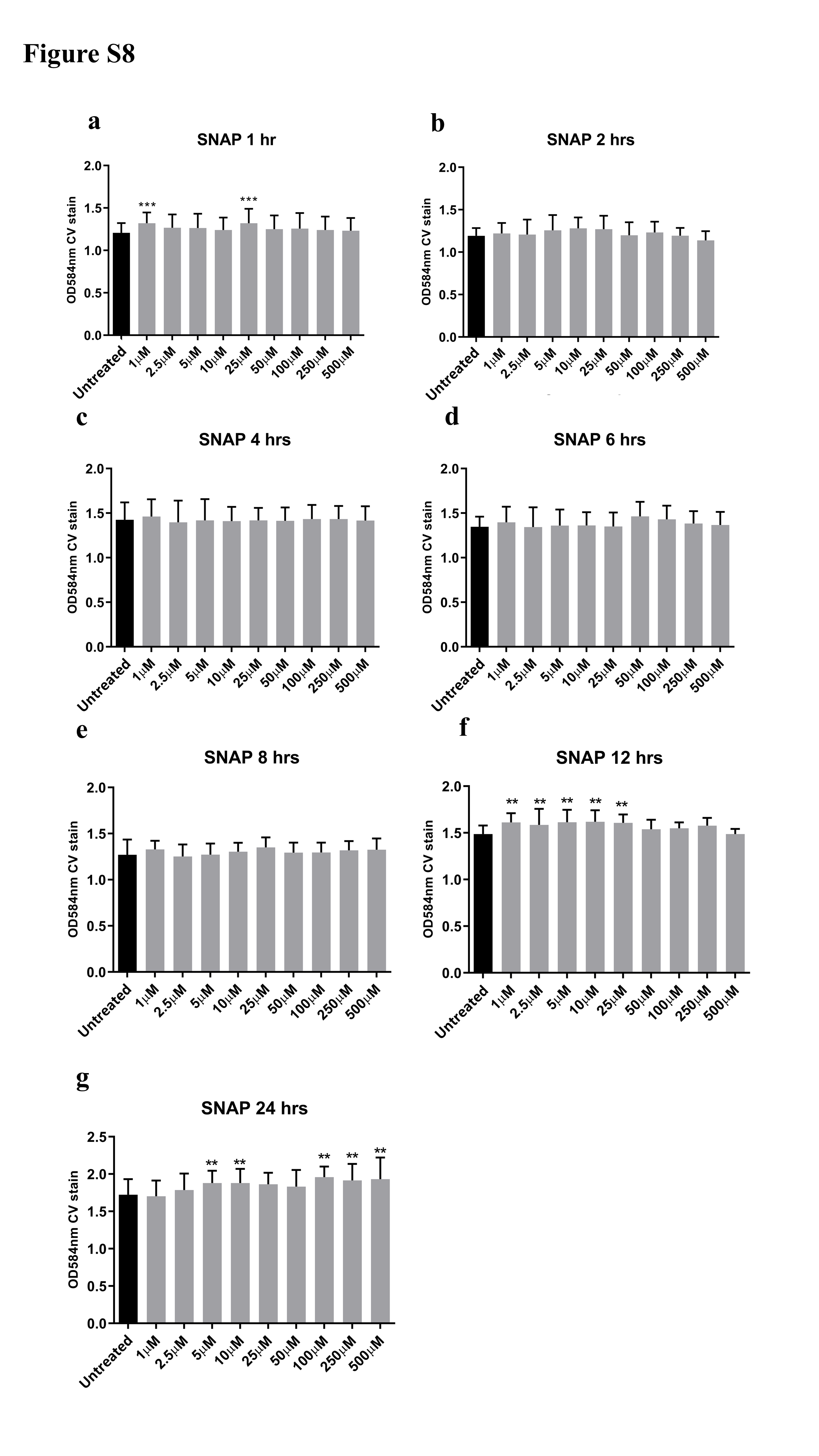

Supplement: Supplementary file 1 — (DOCX 10894 kb) [file 253_2020_10859_MOESM1_ESM.docx]
